# CITCO as an Adjuvant Facilitates CHOP-Based Lymphoma Treatment in hCAR-Transgenic Mice

**DOI:** 10.3390/cells9112520

**Published:** 2020-11-21

**Authors:** Ritika Kurian, William Hedrich, Bryan Mackowiak, Linhao Li, Hongbing Wang

**Affiliations:** 1 Department of Pharmaceutical Sciences, University of Maryland School of Pharmacy, 20 Penn Street, Baltimore, MD 21201, USA; ritika.kurian@umaryland.edu (R.K.); william.hedrich@bms.com (W.H.); bryanmackowiak@umaryland.edu (B.M.); lli@rx.umaryland.edu (L.L.); 2 Pharmaceutical Candidate Optimization, Metabolism and Pharmacokinetics, Bristol-Myers Squibb Company, Princeton, NJ 08540, USA; 3 Laboratory of Liver Diseases, National Institute on Alcohol Abuse and Alcoholism, NIH, Bethesda, MD 20814, USA

**Keywords:** cyclophosphamide, prodrug, CYP2B6/cyp2b10, CAR, CITCO, lymphoma

## Abstract

Non-Hodgkin’s lymphoma (NHL) is a malignant cancer originating in the lymphatic system with a 25–30% mortality rate. CHOP, consisting of cyclophosphamide (CPA), doxorubicin, vincristine, and prednisone, is a first-generation chemotherapy extensively used to treat NHL. However, poor survival rates among patients in advanced stages of NHL shows a need to improve this standard of care treatment. CPA, an integral component of CHOP, is a prodrug that requires CYP2B6-mediated bioactivation to 4-hydroxy-CPA (4-OH-CPA). The expression of CYP2B6 is transcriptionally regulated by the constitutive androstane receptor (CAR, NRi13). We have previously demonstrated that the induction of hepatic CYP2B6 by CITCO, a selective human CAR (hCAR) agonist, results in CHOP’s enhanced antineoplastic effects in vitro. Here, we investigate the in vivo potential of CITCO as an adjuvant of CPA-based NHL treatment in a hCAR-transgenic mouse line. Our results demonstrate that the addition of CITCO to the CHOP regimen leads to significant suppression of the growth of EL-4 xenografts in hCAR-transgenic mice accompanied by reduced expression of cyclin-D1, ki67, Pcna, and increased caspase 3 fragmentation in tumor tissues. CITCO robustly induced the expression of cyp2b10 (murine ortholog of CYP2B6) through hCAR activation and increased plasma concentrations of 4-OH-CPA. Comparing to intraperitoneal injection, oral gavage of CITCO results in optimal hepatic cyp2b10 induction. Our in vivo studies have collectively uncovered CITCO as an effective facilitator for CPA-based NHL treatment with a pharmacokinetic profile favoring oral administration, promoting CITCO as a promising adjuvant candidate for CPA-based regimens.

## 1. Introduction

Lymphomas are solid tumors originating in the immune system and account for 4% of all cancer cases in the United States [1]. In 2018, 74,680 cases of non-Hodgkin’s lymphoma (NHL) were identified, and 50% of NHL patients are above 60 years of age [1,2]. Although NHL has several different subtypes, diffuse large B cell lymphoma (DLBCL) is a commonly occurring aggressive form, representing 31% of all cases [3]. 

Depending on the disease stage and subtype of NHL, various treatment options such as chemotherapy, radiation therapy, and stem cell transplantation have been adopted clinically. Despite the prevalence of multiple treatments, 30% of the NHL patients, do not survive more than five years [4]. To date, the front-line treatment for NHL continues to be the chemotherapeutic regimen composed of cyclophosphamide (CPA), doxorubicin, vincristine, and prednisone (CHOP) [5], given that most third-generation regimens have failed to demonstrate significant improvement over CHOP [6]. The addition of monoclonal antibody rituximab to the CHOP regimen helped improve the median overall survival rate in patients diagnosed with DLBCL [7,8]. Nonetheless, 30–40% of patients will relapse following conventional and salvage chemotherapy regimens largely due to drug resistance and/or intolerable side toxicities [7]. Attenuation of these side-effects requires either dose reduction, dose delay, or treatment withdrawal, but these measures reduce relative dose intensity, thereby impacting patient survival [5]. Therefore, the lack of complete remission and the occurrence of numerous dose-limiting toxicities reinforces the need to enhance the efficacy of this regimen. 

CPA, an alkylating prodrug, is an integral component of CHOP that requires cytochrome P450 (CYP) metabolic bioactivation to generate the cytotoxic metabolite [9]. The critical step in the bioactivation of CPA is the oxidation to form 4-hydroxy-CPA (4-OH-CPA). This hydroxylated derivative of CPA is relatively unstable and exists in equilibrium with its acyclic form, aldophosphamide [9,10]. This intermediate metabolite binds to erythrocytes and is transported to the tumor tissue, whereby the spontaneous process of β elimination generates phosphoramide mustard and acrolein [11,12,13]. Phosphoramide mustard spontaneously cyclizes to aziridinium ion and causes cross-linking of DNA strands leading to cytotoxic effects [9]. Thus, metabolic oxidation of CPA is essential to the formation of phosphoramide mustard. Although multiple CYP enzymes such as CYP2A6, CYP2B6, CYP3A4, CYP3A5, CYP2C9, CYP2C18, and CYP2C19 are involved in the C4 hydroxylation of CPA, CYP2B6 is the predominant enzyme, responsible for ~45% of CPA metabolism [14,15,16]. While most CPA undergoes CYP2B6-mediated bioactivation, a small fraction is subjected to CYP3A4-dependent N-dechloroethylation, resulting in the formation of a neurotoxic metabolite and an inactive byproduct [17,18,19]. Our approach to potentially boost the therapeutic efficacy of CPA is to intensify its metabolic conversion to 4-OH-CPA by selectively inducing CYP2B6. 

CYP2B6 is a highly inducible gene, and its transcriptional activation is regulated by the interaction of nuclear receptor complexes with enhancer sequences found in the CYP2B6 promoter region. The constitutive androstane receptor (CAR, NR1i3) is a liver-enriched transcription factor that plays a predominant role in the induction of CYP2B6 [20,21,22]. Under physiological conditions, CAR is present in the cytoplasm in a complex with different chaperone proteins [23]. Interaction between CAR and its agonist causes dissociation of CAR from the chaperone proteins resulting in its translocation into the nucleus [24]. In the nucleus, the heterodimer complex between CAR and the retinoid-X-receptor (RXR) recognizes response elements in the promoter regions of its target genes that encode for different proteins, including CYP2B6 [21]. CITCO (6-(4-chlorophenyl) imidazo [2.1-b]thiazole-5-carbaldehyde-O-(3,4dichlorobenzyl)oxime) has been established as a selective human CAR (hCAR) activator that preferentially induces the expression of CYP2B6 over CYP3A4 in cultured human primary hepatocytes [25]. Our laboratory has previously demonstrated that CITCO can enhance CHOP’s therapeutic index in a multicellular co-culture model [26]. Although CITCO as an adjuvant for CPA-based regimens appears promising, it is largely unknown whether these in vitro findings could successfully advance to a physiologically relevant in vivo model. Moreover, the preliminary pharmacokinetic (PK) profile of this selective hCAR activator has not been investigated.

This report found that CHOP in conjunction with CITCO has significantly attenuated the growth of lymphoma xenografts in an hCAR-transgenic mouse model. The inclusion of CITCO also repressed the expression of a cluster of genes associated with cell proliferation in CHOP-treated xenografts. Furthermore, oral administration of CITCO is sufficient to robustly induce hepatic expression of cyp2b10 (the murine ortholog of CYP2B6) for CPA activation while maintaining a relatively low systemic exposure. The information gained regarding the PK profile of CITCO will be valuable towards developing this molecule as a potential adjuvant drug candidate to facilitate CPA-based regimens. 

## 2. Materials and Methods

### 2.1. Chemicals and Biological Reagents

CITCO, CPA, doxorubicin, prednisone, vincristine, hexamethyl phosphoramide (HMP), chrysin, 1,4-Bis [2-(3,5-Dichloropyridyloxy)] benzene (TCPOBOP), semicarbazide, ammonium formate, methyl-tert butyl ether (MTBE), isopropyl alcohol (IPA) formic acid (FA) and corn oil were all purchased from Sigma Aldrich (St. Louis, MO, USA). 4-hydroxy cyclophosphamide (4-OH-CPA) was purchased from Toronto Research Chemicals (North York, ON, Canada). Optima LC/MS grade acetonitrile (ACN), methanol (MeOH), water (H_2_O) were obtained from Fisher Scientific (Pittsburg, PA, USA). Sterile normal saline solution was obtained from Vedco (St. Joseph, MO, USA). DL5009 was synthesized at the University of Maryland School of Pharmacy as described previously [27].

### 2.2. Mouse Liver Perfusion and Mouse Primary Hepatocyte/EL-4 Cell Co-Culture

Mouse primary hepatocytes (MPH) were isolated from hCAR-transgenic or wildtype C57BL/6 mice by a two-step collagenase perfusion method with moderate modification [28]. Briefly, the mouse liver was initially perfused with buffer A (1 × Krebs Ringer Hepes Ca^2+^ free buffer containing 526 nM EGTA) for 6–7 min followed with buffer B (1 × Krebs Ringer Hepes Mg^2+^ free buffer containing 40 mg/100 mL collagenase I from Worthington Biochemical Corporation) perfusion for 7–8 min at 8 mL/min buffer flow rate. The hepatocytes were further purified by filtering the cell suspension through the 70 µM nylon cell strainer and low speed centrifuging at 50× *g*, 2 min at 4 °C. The hepatocytes were seeded at 0.5 × 10^6^ cells/well in 12-well BioCoat plates in Dulbecco’s modified Eagle’s medium supplemented with 5% fetal bovine serum, 100 U/mL penicillin, 100 µg/mL streptomycin, 4 µg/mL insulin, and 0.1 µM dexamethasone. For MPH/EL-4 coculture, MPH cultured in collagen-coated 12-well plates at 37 °C in a humidified atmosphere of 5% CO_2_ were pretreated with 0.1% DMSO or 1 µM CITCO for 24 h, followed by the insertion of a 3.0 µm polycarbonate membrane inserts (Sigma-Aldrich, St. Louis, MO, USA). EL-4 cells (0.5 × 10^6^) in suspension were transferred into the insert chamber with a final volume of 2mL supplemented William’s Medium E per well for the MPH/EL-4 coculture. The cocultures were treated to designed concentrations of CHOP in the presence or absence of CITCO as indicated. 

### 2.3. Western Blot 

Total protein was extracted from mouse liver samples and mouse xenograft samples, respectively. Protein samples from different groups were electrophoretically separated on Bis-Tris gels (4–12%) and transferred to polyvinylidene fluoride membranes. Subsequently, membranes were incubated with primary antibodies against CYP2B10 (1:1000) (Cell Signaling Technology, Danvers, MA, USA) or cleaved caspase-3 (1:1000) (Cell Signaling Technology) or β-actin (1:3000) (Sigma Aldrich) at 4 °C overnight. After incubation with horseradish peroxidase secondary antibodies, Radiance Q chemiluminescence substrate (Azure, Dublin, CA, USA) and West Pico chemiluminescent substrate (ThermoFisher, Waltham, MA, USA) were used to develop blots. Densitometry was analyzed using an Azure Biosystems Imager c300. The complete images of all Western Blots were presented as supplementary data (Figure S1).

### 2.4. Real-Time RT-PCR

Total RNA was isolated using TRIzol reagent (ThermoFisher) and reverse-transcribed using a High Capacity cDNA Archive kit (Applied Biosystems, Foster, CA, USA) according to the manufacturer’s instructions. The Real-time PCR assay was performed using SYBR Green PCR Mastermix (Qiagen) on an ABI StepOnePlus Real-Time PCR system (Applied Biosystems). Primer sequences for cyp2b10, cyclin-D1, Ki-67, Pcna and GAPDH are: *Cyp2b10* 5′-TCAGGTGATCGGCTCACAC-3′ (forward) and 5′-CATCCAGGAACTGGTCAGGA-3′ (reverse); *Cyp3a11* 5′-GTCAAACGCCTCTCCTTGCTG-3′ (forward) and 5′-GGCTTGCCTTTCTTTGCCTTC-3′ (reverse); *Cyp3a16* 5′-CTGCAGGAGGAGATCGA-3′ (forward) and 5′-CCATCGCCATCACGGTA-3′ (reverse); zza *cyclin-D1* 5′-AGGCGGATGAGAACAAGCAGA-3′ (forward) and 5′-CAGGCTTGACTCCAGAAGGG-3′ (reverse); *Ki-67* 5′-AATCCAACTCAAGTAAACGGGG-3′ (forward) and 5′-TTGGCTTGCTTCCATCCTCA-3′ (reverse); *Pcna* 5′-TAAAGAAGAGGAGGCGGTAA-3′ (forward) and 5′-TAAGTGTCCCATGTCAGCAA-3′ (reverse); and *Gapdh* 5′-TCCACTCACGGCAAATTCAACG-3′ (forward) and 5′-TAGACTCCACGACATACTCAGC-3′ (reverse). Expression values were quantified using the equation: fold over control = 2^ΔΔCt^ method, where ΔCt represents the differences in cycle threshold numbers between the target gene and GAPDH, and ΔΔCt represents the relative change in these differences between control and treatment groups.

### 2.5. Cell Viability

MPH and EL-4 cells were co-cultured, as described previously [26]. The co-culture was treated with CITCO (1 µM) or vehicle control (0.1% DMSO) 24 h before treatment with CHOP at indicated concentrations. The composition of the four drugs in CHOP was estimated based on previous reports [29]. In brief, 100 µM of CHOP indicates a mixture of CPA (100 µM), doxorubicin (5 µM), vincristine (0.14 µM), and prednisone (10 µM). The viability of EL-4 cells was determined at selected time points with a Cellometer Auto T4 (Nexcelom Biosciences, Lawrence, MA, USA) using trypan blue exclusion. Cell viability was expressed as a percentage of vehicle control (0.1% DMSO).

### 2.6. Xenograft Study

Male hCAR-transgenic C57BL/6 mice (6–8 weeks old) were inoculated subcutaneously (s.c.) with 1 × 10^6^ EL-4 cells suspension in 200 μL PBS in the left and right flanks. The mice were monitored for general condition and tumor growth daily. Tumors were measured three times a week with Vernier calipers, and the tumor volumes were calculated with the equation (l × w^2^)/2. When tumors approach the volumes around 100 mm^3^ (mice with tumors smaller than 70 mm^3^ were not used), the mice were randomly divided into 4 groups (*n* = 7/group). The mice were subsequently treated (via intraperitoneal injection) with corn oil, 20 mg/kg CITCO, 40 mg/kg CHOP, CITCO + CHOP on a schedule as depicted in Figure 3A. CITCO and CHOP were dissolved in corn oil and saline, respectively, and prepared fresh for each treatment. Tumor mass and mouse body weight were monitored 3 times/week. Mice were euthanized 6 days post-end-of-treatment. Tumors were removed, and weights were recorded. Livers were harvested and snap-frozen in liquid nitrogen for molecular examination. All animal studies procedures were approved by the Institutional Animal Care and Use Committee (IACUC) of the University of Maryland School of Medicine. All animal experiments met the Animal Welfare guidelines. Mice were housed in laminar flow cabinets at room temperature with a 24-h night/day cycle and fed with pellets and water *ad libitum*.

### 2.7. Pharmacokinetic Studies 

For CPA and 4-OH-CPA, male hCAR-transgenic C57BL/6 mice (6–8 weeks old) were divided into 2 groups (*n* = 5/group). Group 1 received corn oil on day 1 and 80 mg/kg CHOP on day 2. Group 2 received 20 mg/kg CITCO on day 1 and 80 mg/kg CHOP on day 2. All treatments were delivered intraperitoneally (IP). Serial blood samples were collected via the tail vein at 0.083, 0.25, 0.5, 1, 2, and 4 h using a heparinized capillary tube. The blood was further treated with 0.5 M EDTA. Plasma was separated by centrifugation and transferred to another tube containing 2 M semicarbazide (to stabilize 4-OH-CPA), vortexed, and stored at −80 °C until LC-MS/MS analysis. For CITCO, male C57BL/6 mice (6–8 weeks old) (*n* = 3/group) were dosed with 20 mg/kg CITCO prepared in corn oil by IP or oral gavage (PO). Serial blood samples were collected via the tail vein at 0.083, 0.25, 0.5, 1, 2, 4, 8, 12, and 24 h using a heparinized capillary tube. These blood samples were processed by centrifugation to obtain serum which was stored at −80 °C until LC-MS/MS analysis. In a separate experiment, male C57BL/6 mice (6–8 weeks old) were treated with 20 mg/kg CITCO prepared in corn oil by IP and PO routes. Liver distribution of CITCO was examined approximately 4 h after dosing with liver tissues isolated, snap-frozen, and stored −80 °C until LC-MS/MS analysis. 

### 2.8. LC-MS/MS Quantitative Analysis 

(a) CPA and 4-OH-CPA Sample preparation: 10 µL of the plasma plus semicarbazide solution was aliquoted into a fresh tube. Then, 2 µM hexamethylphosphoramide was added as the internal standard. 40 µL of acetonitrile was added to precipitate proteins, and samples were vortexed for 10 min at room temperature. Following centrifugation, the supernatant was extracted twice with ethyl acetate. The organic phase was evaporated to dryness under a nitrogen stream and reconstituted in the mobile phase. LC-MS/MS analysis was performed on a Waters TQD triple quadrupole mass spectrometer operated with positive electrospray ionization (Waters Corporation, Milford, MA, USA) as described previously [30]. The liquid chromatography (LC) separation was performed on an Agilent Xorbax C18 (100 mm × 2.1 mm, particle size 3.5 µm). The mobile phase consisted of ACN:H_2_O (0.05% TFA) 80:20 *v*/*v*. The flow rate was 0.4 mL/min, with a total run time of 5 min and an injection volume of 10 µL. Using selected reaction monitoring (SRM) mode for mass detection, the following transitions were monitored for CPA (**m/z** 261 → 140), 4-OH-CPA-semicarbazide (*m/z* 334 → 221) and HMP (*m/z* 180 → 135).

(b) CITCO Sample preparation: To 10 µL of mouse serum samples, 100 µL of MeOH containing 40 nM of DL5009, an analog of CITCO, as internal standard, was added. Samples were vortexed and incubated on ice for 10 min. Then, 125 µL of MTBE was added, and samples were incubated for 1 h on ice with occasional stirring. After that, 125 µL of ice-cold H_2_O was added to each sample. The samples were briefly vortexed, followed by incubation on ice for 15 min. Following this incubation, the samples were centrifuged at 3000 rpm for 5 min, and the supernatant was transferred to HPLC glass vial inserts. Then, 125 µL of MTBE was added to the remaining aqueous layer and incubated on ice for another 15 min with occasional stirring. These samples were also centrifuged at 3000 rpm for 5 min. This supernatant was combined with the previously obtained supernatant. The organic phase was evaporated to dryness under a nitrogen stream and reconstituted with 50 µL 50:40:10:0.1 IPA:acetonitrile:H_2_O: FA. Authentic calibration standards ranging from 5–5000 nM were prepared in 50:40:10:0.1 IPA:acetonitrile:H_2_O: FA, quality control samples were prepared in 50:40:10:0.1 IPA:acetonitrile:H_2_O:FA and in mouse serum (15–4000 nM). LC-MS/MS analysis was performed on a TQ-XS 2 triple quadrupole mass spectrometer coupled to an Acquity UPLC platform (Waters Corporation). The LC separation was performed on an Acquity UPLC BEH HILIC (100 × 2.1 mm, 1.7 µm) (Milford, MA, USA) operated at 30 °C. Solvent A and B consisted of 10 mM Ammonium Formate in 100:0.1 H_2_O:FA and 10 mM Ammonium Formate in 10:90:0.1 H_2_O:ACN:FA, respectively. The gradient program was 0.00–2.00 min, 100% B; 2.25–3.75 min, gradient to 10% B; 3.75–4.00, gradient to 100% B; 4.00–5.00, 100% B. The flow rate was 0.3 mL/min, and the injection volume was 2 µL. Tandem mass spectrometry was performed in the positive-ion mode, and the electrospray ionization source parameters were as follows: capillary voltage, 3.5 KV; cone voltage, 52 KV; desolvation gas flow, 600 L/hr; source temperature 150 °C. SRM was used for mass detection with the following transitions, CITCO (*m/z* 436.1 → 260.04) and 5009 (*m/z* 384.18 → 276.12). Data collection and analysis were carried out by MassLynx (Waters Corporation).

### 2.9. Statistics

Data were expressed as Mean ± SD or Mean ± SEM. Statistical comparisons were made using one-way and two-way ANOVA with a Bonferroni post-test and also with unpaired, one-tailed, and two-tailed Student t-test with a Mann–Whitney post-test where appropriate. Statistical significance was set at *: *p* < 0.05, **: *p* < 0.01. 

## 3. Results

### 3.1. CITCO Increases CHOP-Mediated Cytotoxicity in EL-4 Cells Cocultured with hCAR-TG-MPH but Not WT-MPH

**Figure 1:**
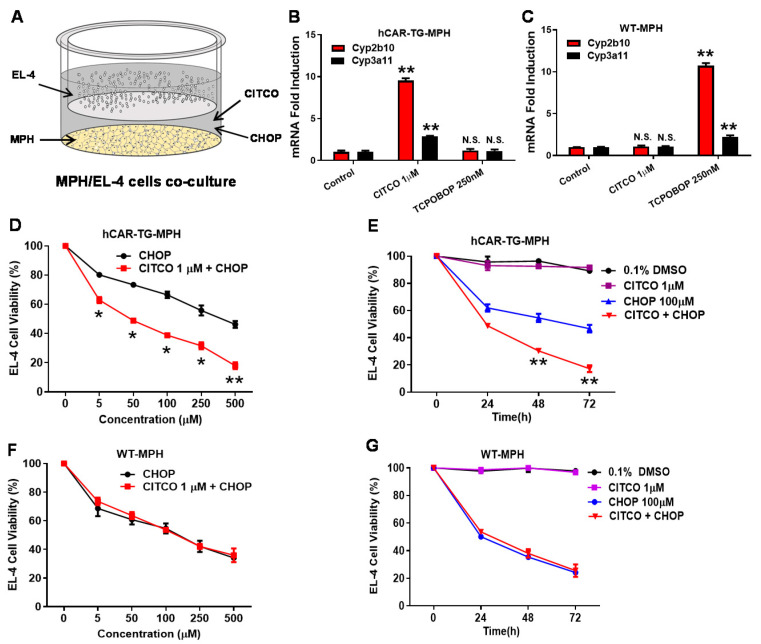
CITCO increases CHOP-mediated cytotoxicity in EL-4 cells co-cultured with hCAR-TG-MPH, not WT-MPH. (**A**) Schematic depiction of the co-culture model. (**B**,**C**) Cultured hCAR-TG-MPH and WT-MPH were treated with vehicle control (0.1% DMSO), CITCO (1 µM), or TCPOBOP (250 nM) for 24 h. The expression of *Cyp2b10* and *Cyp3a11* was measured by RT-PCR. (**D**,**E**). The co-culture of EL-4/hCAR-TG-MPH was treated with vehicle control (0.1% DMSO), CITCO (1 µM), CHOP at indicated concentrations, or CITCO + CHOP in a concentration- or time-dependent manner. EL-4 cell viability was measured as indicated in Materials and Methods. (**F**,**G**). The co-culture of EL-4/WT-MPH was treated as described above, and EL-4 cell viability was monitored. Data represent Mean ± SD from three independent experiments as a percentage of vehicle control. *, *p* < 0.05; **, *p* < 0.01, N.S., not significant.

A mouse lymphoma EL-4 cell line derived from a C57BL/6 mouse was used as a murine lymphoma model for studies in hCAR-TG mice. First, we tested the pharmacological responses of EL-4 cells to CITCO and CHOP in primary hepatocytes derived from hCAR-TG (hCAR-TG-MPH) or wildtype (WT) mouse (WT-MPH) co-cultured with EL-4 cells as depicted in Figure 1A. As expected, significant *Cyp2b10* gene induction was observed in hCAR-TG-MPH treated with CITCO but not TCPOBOP, a selective mCAR activator [31] and vice versa in WT-MPH (Figure 1B,C). In contrast, activation of CAR by CITCO and TCPOBOP only led to moderate induction of *Cyp3a11*, the mouse version of CYP3A4. Importantly, in the EL-4/hCAR-TG-MPH co-culture, CITCO markedly enhanced CHOP-mediated EL-4 cytotoxicity both in a concentration- and time-dependent manner, while CITCO alone did not affect EL-4 cell viability (Figure 1D,E). In contrast to this observation, CITCO did not alter CHOP’s anticancer activity in the EL-4/WT-MPH co-culture (Figure 1F,G). Thus, these results indicated that EL-4 cells are sensitive to CHOP-based regimens, and the hCAR-TG model is required to illustrate the therapeutic benefit of CITCO. 

### 3.2. CITCO Enhances CYP2B10-Mediated Activation of CPA in hCAR-TG Mice

**Figure 2:**
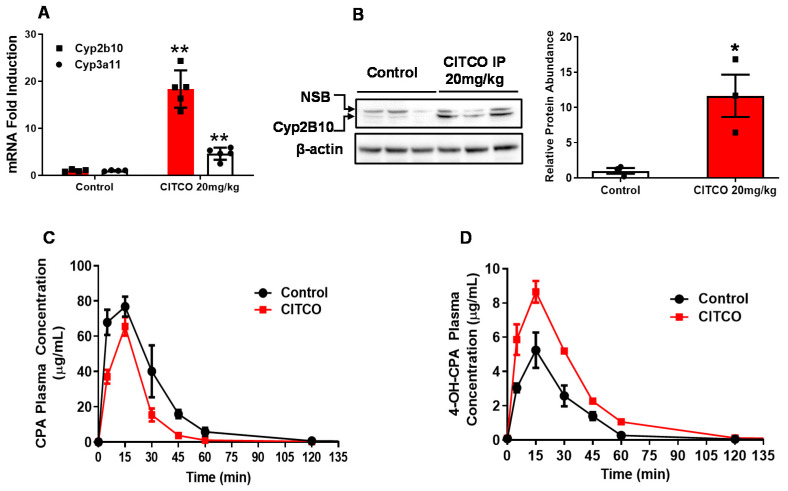
CITCO induces cyp2b10 expression and CPA bioactivation in hCAR-TG mice. hCAR-TG-mice were treated with vehicle control (corn oil) or CITCO (20 mg/kg body weight, IP), and livers were harvested for the detection of *Cyp2b10* and *Cyp3a11* mRNA (**A**) and CYP2B10 protein (**B**) expression. For PK studies, hCAR-TG-mice were pretreated with CITCO (20 mg/kg body weight, IP) for 24 h, followed by a single treatment with CHOP (80 mg/kg body weight, IP). Plasma concentrations of CPA (**C**) and 4-OH-CPA (**D**) over time were measured using LC-MS/MS methods. CPA and 4-OH-CPA values represent the mean ± SEM of 3 LC-MS/MS measurements. NSB: non-specific band. *, *p* < 0.05; **, *p* < 0.01.

In order to assess whether CITCO effectively increases the metabolic bioactivation of CPA in vivo, hCAR-TG mice were treated with CITCO (20 mg/kg, IP) as detailed in Section 2. As shown in Figure 2A,B, CITCO at this dose significantly induced hepatic expression of cyp2b10 mRNA and protein by 20- and 10-fold, respectively, compared to the vehicle control group (corn oil). Comparatively, CITCO induced expression of *Cyp3a11* mRNA was moderate. Pretreatment of hCAR-TG mice with CITCO for 24 h resulted in greater conversion of CPA to 4-OH-CPA as indicated by plasma concentrations of CPA (Figure 2C) and 4-OH-CPA (Figure 2D) over time. Specifically, CITCO lowers the AUC and C_max_ of CPA from 42.9 ± 7.6 to 24.5 ± 4.8 µg/mL.h and 81.3 ± 10.9 to 65.5 ± 11.8 µg/mL, respectively, while increasing the AUC and C_max_ of 4-OH-CPA from 2.8 ± 0.8 to 4.9 ± 1.2 µg/mL.h and 5.4 ± 2.0 to 9.3 ± 1.2 µg/mL, respectively. This led to a higher metabolite: parent AUC ratio in the presence of CITCO (Table 1). Thus, these data suggest that administration of CITCO can achieve significant cyp2b10 induction in the liver, which enhances the metabolic bioactivation of CPA, thereby altering the PK of both CPA and its metabolite, 4-OH-CPA in hCAR-TG mice.

### 3.3. CITCO Improves the Anticancer Activity of CHOP in EL-4 Cell-Derived Xenografts in hCAR-TG Mice

**Figure 3:**
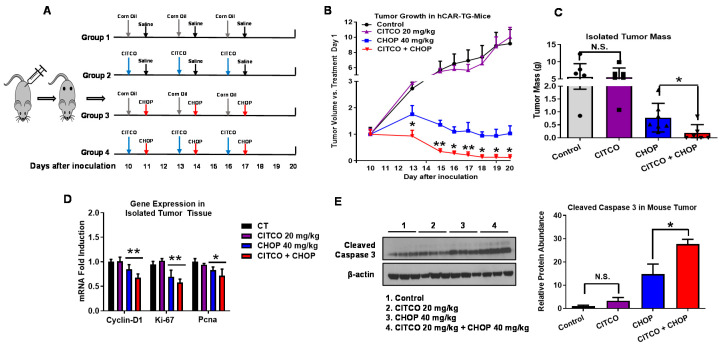
CITCO improves the anticancer activity of CHOP against EL-4 xenograft in hCAR-TG-mice. hCAR-TG-mice inoculated with EL-4 cells for xenograft formation as detailed in Section 2 were divided into 4 groups and treated with vehicle control (corn oil), CITCO (20 mg/kg, IP), CHOP (40 mg/kg, IP) or CITCO + CHOP as depicted in (**A**). Tumor volumes (**B**) from different groups were measured for 20 days post cell injection as indicated. At the end of experiment, tumor weights (**C**) were also measured before stored at −80 °C. mRNA expression of Cyclin D1, ki67, and Pcna was detected using RT-PCR (**D**). Cleaved caspase 3 was measured using Western blotting (**E**). Data represent mean ± SD, *n* = 3–7. *, *p* < 0.05; **, *p* < 0.01, NS, not significant.

EL-4 cells (1 × 10^6^) were inoculated (s.c.) in hCAR-TG male mice to determine the role of hCAR on CHOP-based chemotherapy in vivo, as detailed in Section 2. Nine days after inoculation, the mice were randomly divided into four groups (*n* = 7) and treated via IP with vehicle control (corn oil), CITCO (20 mg/kg), CHOP (40 mg/kg), or CITCO + CHOP, as indicated in Figure 3A. Tumor growth was monitored for 20 days post cell implantation. Our results showed that EL-4 lymphoma cells, syngeneic to C57BL/6 mouse, thrive in the hCAR-TG mice, and CITCO alone did not affect the growth of the EL-4 xenograft (Figure 3B,C). Importantly, compared to the CHOP treatment group, the inclusion of CITCO significantly reduced the average tumor volume and mass. Individual tumor volume changes were presented in Table S1. Accompanied by these pathological changes, the addition of CITCO to CHOP decreased the expression of several cell proliferation markers such as cyclin-D1, Ki67, and Pcna in the EL-4 xenografts (Figure 3D). The expression of cleaved caspase-3, on the other hand, was significantly increased in tumors from hCAR-TG mice treated with CITCO + CHOP compared to those that received CHOP alone (Figure 3E). Moreover, the addition of CITCO did not negatively impact the mice’s body weight, as indicated in Figure S2. Therefore, consistent with our findings in vitro, these data collectively indicate that CITCO when used in combination with CHOP improves its anticancer activity against lymphoma xenograft tumors in hCAR-TG mice. 

### 3.4. Oral Administration of CITCO Effectively Induces Hepatic CYP2B10 Expression

**Figure 4:**
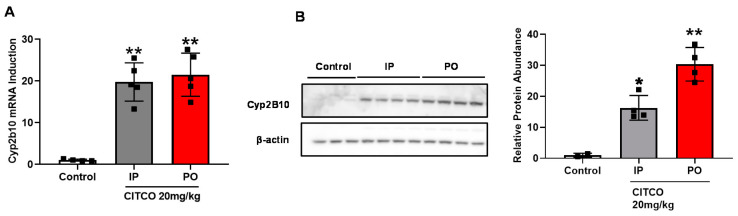
Oral administration of CITCO effectively induces hepatic expression of CYP2B10. hCAR-TG-mice were treated with vehicle control (corn oil) or CITCO at 20 mg/kg via IP or PO as detailed in Section 2. Expression of *Cyp2b10* in the liver was measured by RT-PCAR (**A**) and Western blotting (**B**). Data represent the mean ± SD, *n* = 4–5. *, *p* < 0.05; **, *p* < 0.01.

To this end, our proof-of-concept in vivo studies made use of CITCO administered by the IP route. Next, we examined whether oral administration of CITCO was effective in hepatic cyp2b10 induction. hCAR-TG mice were treated with CITCO at 20 mg/kg/body weight by single gavage (PO) or IP delivery route. CITCO administered by both IP and PO routes at the same dose resulted in effective *Cyp2b10* mRNA induction of 19- and 21-fold over control, respectively (Figure 4A). Notably, CITCO by PO administration led to higher CYP2B10 protein expression in the liver compared to the IP route (Figure 4B). These results suggest that CITCO can be an effective liver enzyme inducer when delivered orally, a clinically convenient dosing route.

### 3.5. CITCO Is Preferentially Directed to Its Site of Action by PO Route 

**Figure 5:**
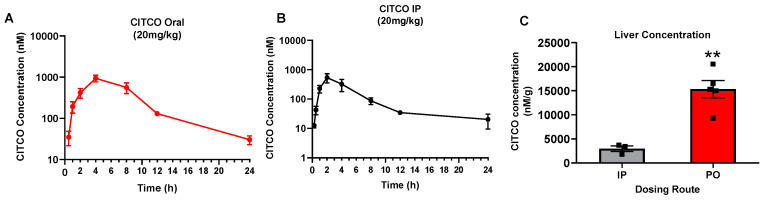
Oral administration of CITCO results in preferential hepatic disposition. hCAR-TG-mice were treated with CITCO at 20 mg/kg via IP or PO as detailed in Section 2. Plasma concentrations of CITCO after PO (**A**) and IP (**B**) were measured using LC-MS/MS analysis at the indicated time points. CITCO concentrations in the liver tissues (**C**) were detected 4 h post-IP or PO administration. Data represent the mean ± SEM, *n* = 3/group. **, *p* < 0.01.

As a potential adjuvant candidate to facilitate CYP2B-mediated CPA bioactivation, the liver is the site of action for CITCO. Subsequently, we investigated its pharmacokinetics (PK) after PO and IP administration. hCAR-TG mice, when dosed with CITCO via the PO and IP routes, revealed PK profiles that exhibit typical characteristics of PO and IP administration, as illustrated in Figure 5A,B, respectively. PK parameters of CITCO as listed in Table 2 suggest that this hCAR activator has a favorable PK profile with a reasonable T_1/2_ between 4–10 h and C_max_ ranging between 500–1000 nM/mL upon PO and IP administration. Since the primary action of CITCO is to activate hCAR and induce CYP2B expression, which is predominantly located in the liver, it is important to determine the hepatic CITCO abundance. CITCO liver concentrations measured in hCAR-TG mice at approximately 4 h following IP and PO administration indicate greater absorption of CITCO in the liver after PO dosing compared to IP (Figure 5C). Collectively, these PK profiles of CITCO help illustrate that CITCO is well tolerated in hCAR-TG mice by both delivery routes; and the proclivity of CITCO to exhibit greater liver accumulation following PO dosing helps its primary goal of hCAR activation, thereby enhancing cyp2b10 induction and CPA bioactivation. 

## 4. Discussion

NHL is the 7th most common cancer in adults in the United States with diverse clinical outcomes [32]. The CHOP regimen continues to be considered as the primary chemotherapeutic choice for the treatment of NHL. However, this widely used multidrug regimen is associated with metabolism/transport-related drug resistance and several dose-limiting toxicities that restrict its use, especially in people over 60 years of age. Hence, there is a need to enhance the efficacy of CHOP-based treatment. CPA, a primary CHOP component and an alkylating prodrug, requires hepatic bioactivation to exert its cytotoxic effect. We have previously shown that CITCO selectively induces CYP2B6, which helps enhance the therapeutic index of CHOP in vitro [26,30]. This study demonstrates that CITCO robustly induces hepatic cyp2b10-mediated metabolic bioactivation of CPA through activation of hCAR in an hCAR-TG mouse model. Importantly, the inclusion of CITCO markedly enhanced CHOP-based attenuation of the growth of lymphoma xenografts in hCAR-TG mice, accompanied by reduced expression of cyclin-D1, Ki67, and Pcna, as well as increased fragmentation of caspase 3. Moreover, we show that oral administration represents an optimal route delivering CITCO to its intended site of action, being the liver, while maintaining low systemic exposure.

Human and mouse CAR exhibit dramatic pharmacological distinctions with only 71% amino acid homology in their ligand-binding domain [33]. CITCO has been known to activate hCAR potently but does not affect mouse CAR (mCAR) [25,34]. In contrast, TCPOBOP efficiently binds and activates mCAR but not hCAR [31,35]. Thus, hCAR-TG mice offer a physiologically relevant system for the investigation of the biological function of hCAR in vivo. The hCAR-TG mouse line used in this study was generated by integrating the 8.5-kb hCAR gene and its 73-kb upstream and 91-kb downstream human genomic DNA into the C57BL/6 mouse genome with an mCAR-knockout genetic background [36]. In co-cultures of mouse primary hepatocytes with EL-4 cells, a murine lymphoma cell line derived from a C57BL/6 mouse, we found that CITCO markedly intensified CHOP-mediated anticancer activity in EL-4 cells co-cultured with MPH from hCAR-TG mice but not from its wildtype littermates. These findings are consistent with previous observations in human primary hepatocytes [26], emphasizing the dependence of CITCO function on hCAR, and support that CITCO-induced hepatic CYP2B10 is sufficient to enhance CPA bioactivation. 

The metabolic conversion of CPA to 4-OH-CPA by hepatic drug-metabolizing enzymes led by CYP2B (CYP2B6 in human and CYP2B10 in mouse) is the initial and rate-limiting step of CPA bioactivation [14,16]. Plasma concentrations of 4-OH-CPA are often used as a biomarker for monitoring the rate of CPA bioactivation and therapeutic efficacy. Our results reveal that CITCO administration robustly induced *Cyp2b10* gene expression in the liver of hCAR-TG mice, which further led to reduced plasma concentration of the parent CPA while it increased the AUC and Cmax levels of 4-OH-CPA, a pharmacokinetic alteration that favors CPA-based chemotherapy. In the lymphoma xenograft study, we found that EL-4 cells, syngeneic to C57BL/6 mice, form rapidly growing xenografts that are pharmacologically responsive to CHOP-based treatment in hCAR-TG mice. Notably, the effects of CHOP on EL-4 xenograft growth was further potentiated by CITCO, resulting in significant tumor shrinkage. In contrast, treatment with CITCO alone was unable to slow down tumor growth, suggesting that CITCO by itself lacks a therapeutic effect; but it can facilitate CPA-based chemotherapeutics’ efficacy.

Excessive cell proliferation has been considered a hallmark of cancer cells, while a decrease in cell proliferation and/or an increase in programmed cell death is typically involved with suppression of neoplastic progression. Almost all cytotoxic chemotherapeutic drugs arrest actively dividing cells either in the G1 or S phase by causing DNA damage or target proliferation signature gene products [37]. Therefore, evaluating the expression of key cell proliferation markers such as the cell-cycle-regulated gene, cyclin-D1, the nuclear protein, Ki67, and Pcna is beneficial to understand the mechanism behind tumor growth suppression. Apoptosis, or type I programmed cell death, is an evolutionarily conserved cell suicide mechanism occurring downstream of DNA damage. CPA, similar to other alkylating agents, renders tumor cells nonviable by inducing apoptosis. Activation of effector caspases such as caspase-3, ensures the process of apoptosis. In this study, we found that CITCO promotes CHOP-mediated attenuation of cell proliferation markers and augmentation of the expression of cleaved caspase-3 in treated EL-4 xenografts, further elaborating the therapeutic benefits of concomitant use of CITCO with CHOP in lymphoma chemotherapy. 

Structurally, CITCO consists of different hydrophobic functional groups resulting in poor aqueous solubility, thereby limiting possibilities for intravenous administration. On the other hand, IP delivery though convenient in animal studies is not preferred clinically. Poorly soluble drugs are quite often administered via the oral route. Hence it is important to investigate the potential of CITCO to activate hCAR and induce cyp2b10 when delivered orally [38]. Our results show that hepatic cyp2b10 expression in hCAR-TG mice was drastically induced by CITCO when dosed orally. Comparison of the PK behaviors of CITCO after IP and PO administration further reveals that while both IP and PO exhibited relatively low but adequately stable systemic exposure of CITCO with the T_1/2_ ranging between 4–10 h, PO resulted in significantly higher liver accumulation of CITCO. Given that the liver is the target of action for CITCO where CAR and CYP2B10 are primarily localized, this unique hepatic preferential accumulation accompanied with low systemic level favors CITCO as an adjuvant remedy for CPA bioactivation and CPA-containing chemotherapy. While it is well-known that orally delivered drugs tend to undergo the first-pass metabolism, the hydrophobic nature and physicochemical properties of CITCO may enable it to be preferentially directed to the liver, which facilitates hCAR activation [39]. 

Collectively, our findings show that the inclusion of CITCO in the CHOP regimen significantly enhances its therapeutic efficacy on NHL treatment. This multidrug chemotherapeutic regimen’s improved activity could be attributed to the increased metabolic bioactivation of CPA to 4-OH-CPA; hence, augmenting the cytotoxic effect of this alkylating prodrug. Evaluating the in vivo features of CITCO reveal that it can be effective when delivered by an oral route, which led to extensive absorption of CITCO in the liver with relatively low blood concentration and comparably low non-specific side effects. Meanwhile, we realize the limitations of the current study, including the relatively narrow beneficial effects of CITCO on CPA only, without adequately considering other components, such as vincristine and doxorubicin. Future more intensive studies are warranted to determine the effective dose, metabolic stability, and toxicity profile of CITCO. However, our current findings provide proof-of-concept support for the use of CITCO as a promising adjuvant drug candidate to facilitate CPA-based chemotherapy. 

## Figures and Tables

**Table 1:** Pharmacokinetic parameters of CPA and 4-OH-CPA after CITCO pretreatment in hCAR-TG-mice.

CPA (Mean ± SD)				4-OH-CPA (Mean ± SD)		
**Parameters**	**−CITCO**	**+CITCO**	***p* Value**	**−CITCO**	**+CITCO**	***p* Value**
AUC_0-last_ (µg/mL·h)	42.9 ± 7.6	24.5 ± 4.8	0.0014	2.8 ± 0.8	4.9 ± 1.2	0.020
C _max_ (µg/mL)	81.3 ± 10.9	65.5±11.8	0.0062	5.4 ± 2.0	9.3 ± 1.2	0.005
**Metabolite: Parent AUC Ratio**		**−CITCO**		**+CITCO**	***p* Value**	
		0.070 ± 0.018		0.199 ± 0.06	0.015	

**Table 2:** CITCO Pharmacokinetic parameters from hCAR-TG-mice dosed 20mg/kg via PO and IP route.

Parameters	CITCO (Mean ± SEM)	
	PO	IP
AUC_0-24_ (nmol/ml*h)	7070 ± 1371	2716 ± 769
AUC_0-∞_ (nmol/ml*h)	7252 ± 1367	3145 ± 591
C_max_ (nmol/ml)	937 ± 170	541 ± 186
T_max_ (h)	4.00	2.00
T_½_ (h)	4.07 ± 0.307	9.33 ± 5.13

## Supplementary Materials

The following are available online at https://www.mdpi.com/2073-4409/9/11/2520/s1, Figure S1: Complete images of the Western blotting; Figure S2: Mouse body weight changes during the xenograft study; Table S1: Individual tumor volume changes during the xenograft study.
