# Supplementary material for: CITCO as an Adjuvant Facilitates CHOP-Based Lymphoma Treatment in hCAR-Transgenic Mice
Source: Cells. 2020 Nov 21;9(11):2520. doi: 10.3390/cells9112520 (PMC7700167; doi:10.3390/cells9112520)
Supplement: Supplementary file 1 [file cells-09-02520-s001.pdf]

cells

MDPI

CITCO As an Adjuvant Facilitates CHOP-based Lymphoma Treatment in hCAR-transgenic Mice

Ritika Kurian [1 £], William D. Hedrich[1,2 £], Bryan Mackowiak[1,3], Linhao Li[1], and Hongbing Wang[1,*]

[1] Department of Pharmaceutical Sciences, University of Maryland School of Pharmacy, 20 Penn Street, Baltimore, MD, USA

[2] Current Address: Pharmaceutical Candidate Optimization, Metabolism and Pharmacokinetics, Bristol-Myers Squibb Company, Princeton, NJ, USA

[3] Current Address: Laboratory of Liver Diseases, National Institute on Alcohol Abuse and Alcoholism, NIH, Bethesda, MD, USA

[£] Ritika Kurian and William D. Hedrich contributed equally to this work.

[*] Correspondence: Hongbing Wang; Department of Pharmaceutical Sciences, University of Maryland School of Pharmacy, 20 Penn Street, Baltimore, MD 21201, hongbing.wang@rx.umaryland.edu Tel.: (410-706-1280)

Figure-S1. Complete images of the Western blotting.

Figure-S2. Mouse body weight changes during the xenograft study

Table-S1. Individual tumor volume changes during the xenograft study

Figure 2: Panel B

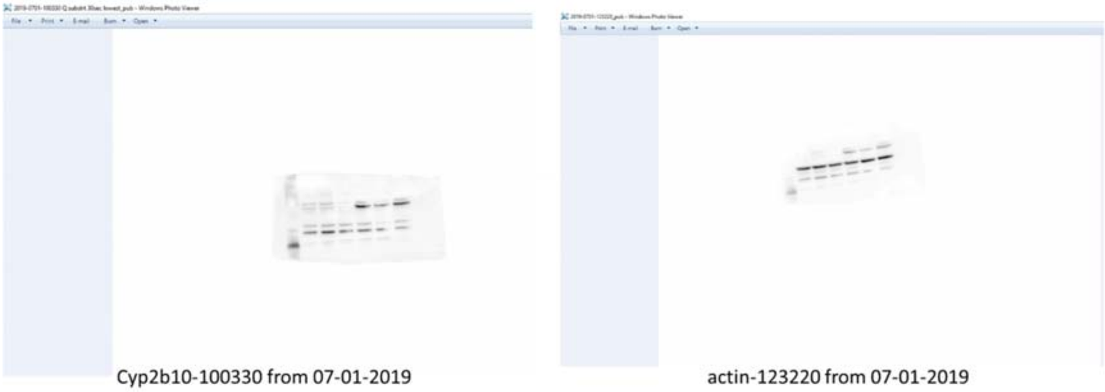

Cyp2b10-100330 from 07-01-2019 actin-123220 from 07-01-2019

Figure 3: Panel E

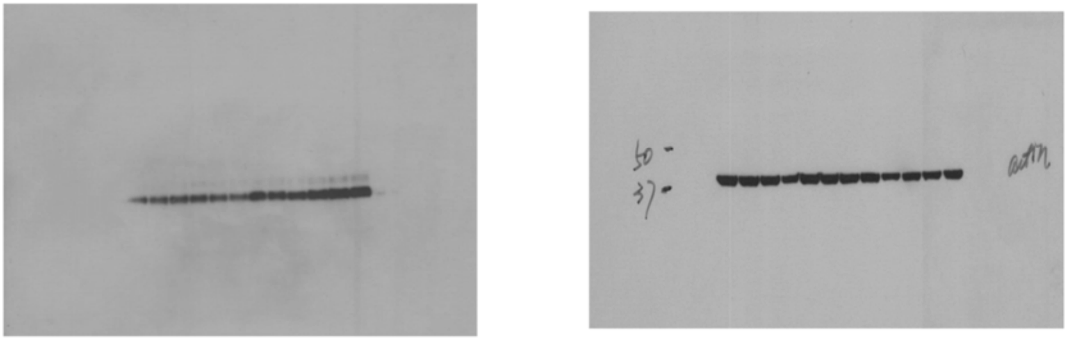

Cleaved Caspase-3 Actin

Figure 4 : Panel B

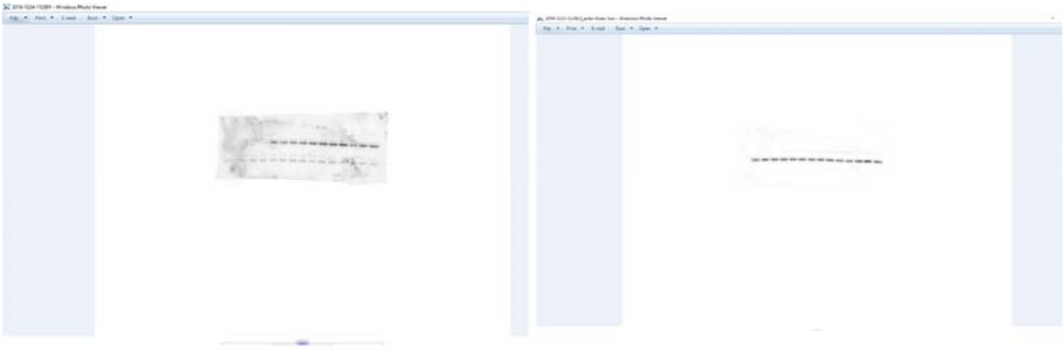

Cyp2b10-112801 from 12-24-2019 actin-122423 from 12-24-2019

Figure-S1. Complete images of the Western blotting.

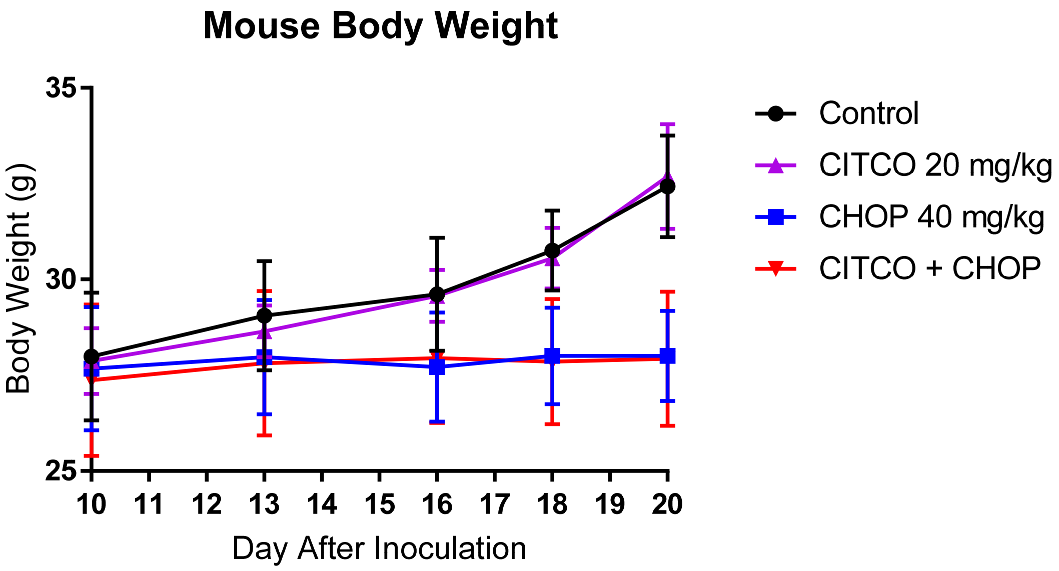

Figure-S2. Mouse body weight changes during the xenograft study.

Table-S1. Individual tumor volume changes during the xenograft study

Treatment Group	Mouse #	Day 10	Day 13	Day 15	Day 16	Day 17	Day 18	Day 19	Day 20
**1 (Control)**	1A	148	385	1091	1203	1250	1303	1517	1561
	1B	110	408	425	470	500	527	633	678
	1C	123	324	650	707	762	825	1025	1230
	1D	162	406	431	538	566	599	808	851
	1E	228	789	1378	1737	1830	1935	2108	1281
	1F	172	281	1646	1844	1917	2020	2569	2648
	1G	90	217	426	536	576	617	759	804
**2 (CITCO)**	2A	110	417	1027	1041	1157	1568	1766	2294
	2B	154	427	722	751	920	1223	1332	1805
	2C	101	407	455	428	526	706	800	957
	2D	132	351	371	366	438	506	643	841
	2E	209	354	902	865	947	1128	1390	1709
	2F	201	364	1132	1101	1249	1908	2188	2382
	2G	126	439	1163	1117	1269	1698	1921	2389
**3 (CHOP)**	3A	160	320	271	204	234	220	202	245
	3B	108	208	182	142	152	129	123	131
	3C	197	345	198	142	161	135	129	138
	3D	128	257	229	176	178	127	136	175
	3E	217	418	299	87	92	72	73	77
	3F	194	236	201	159	164	149	150	158

	3G	86	133	110	87	92	72	73	77
**4 (CITCO + CHOP)**	4A	142	121	79	64	60	42	39	38
	4B	147	126	69	60	59	53	54	51
	4C	129	104	21	16	15	0	0	0
	4D	177	157	104	74	60	54	49	48
	4E	191	128	0	0	0	0	0	0
	4F	180	168	66	41	37	0	0	0
	4G	119	69	0	0	0	0	0	0

Tumor volume measurements of individual mice in xenograft study. Day indicated is days post-inoculation with EL-4 cells. Volumes presented as mm^3 as determined with the equation $(l \times w^2)/2$.
